# Enhanced Photoelectrochemical Water Splitting of In_2_S_3_ Photoanodes by Surface Modulation with 2D MoS_2_ Nanosheets

**DOI:** 10.3390/nano14201628

**Published:** 2024-10-11

**Authors:** Roshani Awanthika Jayarathna, Jun-Ho Heo, Eui-Tae Kim

**Affiliations:** 1 Department of Materials Science & Engineering, Chungnam National University, Daejeon 34134, Republic of Korea; rajayara@tec.rjt.ac.lk (R.A.J.); ppi6140@cnu.ac.kr (J.-H.H.); 2 Department of Materials Technology, Faculty of Technology, Rajarata University of Sri Lanka, Mihintale 50300, Sri Lanka

**Keywords:** photoelectrochemistry, water splitting, photoanodes, indium sulfide, molybdenum disulfide

## Abstract

Photoanodes with ample visible-light absorption and efficient photogenerated charge carrier dynamics expedite the actualization of high-efficiency photoelectrochemical water splitting (PEC-WS). Herein, we fabricated the heterojunction nanostructures of In_2_S_3_/MoS_2_ on indium-doped tin oxide glass substrates by indium sputtering and sulfurization, followed by the metal–organic chemical vapor deposition of 2D MoS_2_ nanosheets (NSs). The photocurrent density of In_2_S_3_/MoS_2_ was substantially enhanced and higher than those of pristine In_2_S_3_ and MoS_2_ NSs. This improvement is due to the MoS_2_ NSs extending the visible-light absorption range and the type-II heterojunction enhancing the separation and transfer of photogenerated electron–hole pairs. This work offers a promising avenue toward the development of an efficient photoanode for solar-driven PEC-WS.

## 1. Introduction

Photoelectrochemical water splitting (PEC-WS) is propitious to produce hydrogen (H_2_) to satisfy the world’s energy demands and environmental challenges since H_2_ gained its importance as an ideal carbon-free energy carrier and an alternative to fossil fuels in addition to its key roles in hydrogenation, petroleum refineries, and fertilizers [1,2]. However, the slow anode oxygen evolution reaction (OER) impedes the applicability of PEC-WS on a large scale [1]. As a solution, semiconductor photoanodes have gained research attention and become popular in solar energy conversion.

In_2_S_3_, an n-type semiconductor, has attracted considerable attention due to its relatively narrow band gap of 2.0–2.3 eV for visible-light utilization, high photosensitivity, and chemical stability [3,4]. However, pristine In_2_S_3_ shows a relatively low PEC efficiency owing to its fast charge recombination inside the bulk and on the surface. Li et al. [5] reported β-In_2_S_3_ nanosheets (NSs) with a photocurrent density of 35.7 μA/cm^2^. Yao et al. [6] showed a PEC performance around 15 μA/cm^2^ by In_2_S_3_ NSs arrays. The formation of a heterojunction with an appropriate semiconductor can effectively minimize this drawback, resulting in improved charge separation and transfer and enhanced optical absorption.

Among the semiconductors that form favorable energy band alignments with In_2_S_3_, 2D-layered MoS_2_ can be a promising candidate because of its tunable bandgap energy, excellent photoexcitation, good chemical stability, and earth abundance [7,8]. It also exhibits tunable bandgaps from ~1.2 eV for the indirect gap of the bulk form to ~1.9 eV for the direct gap of the monolayer and a relatively high mobility (a few hundred cm^2^/Vs) [8,9,10]. The photoelectrochemical (PEC) activity of 2D MoS_2_ is also strongly affected by its architecture standing vertically on the substrate, which provides additional conductive channels for photoexcited carriers [11].

Information on the PEC-WS of In_2_S_3_ heterojunctioned with vertically-standing 2D MoS_2_ NSs is limited. Singh et al. [12] reported the photocatalytic reaction of In_2_S_3_ functionalized with MoS_2_ nanoflowers. Liu et al. [13] showed that MoS_2_ nanodot-decorated In_2_S_3_ nanoplates can be applied for PEC but at a low photocurrent level of 1 μA cm^−2^. Sun et al. [14] later successfully applied a one-pot strategy for growing In_2_S_3_/MoS_2_ with an anodic photocurrent of 0.06 mAcm^−2^ at 0.341 V vs. RHE; nevertheless, the performance still has room for improvement. In the present study, we report vertical 2D MoS_2_ NSs on In_2_S_3_ nanoparticles (NPs) as an alternative anodic choice to OER for significantly improved PEC-WS. The heterojunction effect of In_2_S_3_/MoS_2_ was demonstrated through systematic PEC analysis and photo-excited carrier transfer properties across In_2_S_3_/MoS_2_.

## 2. Materials and Methods

In_2_S_3_ was synthesized on indium-doped tin oxide (ITO) glass substrates via sputtering at 30 W power under a pressure of 3 mTorr for 40 s, followed by sulfurization under a H_2_S flow rate of 200 standard cubic centimeters per minute (SCCM) at 300 °C for 30 min under a pressure of 10 Torr. MoS_2_ NSs were then decorated on the In_2_S_3_/ITO and bare ITO substrates at 300 °C for 8 min under a pressure of 1 Torr by using a metal–organic chemical vapor deposition (MOCVD) system with Mo (CO)_6_ and H_2_S gas (5 vol. % in balance N_2_) as Mo and S precursors, respectively. Mo (CO)_6_ was vaporized at 20 °C and delivered into a quartz tube using Ar gas of 20 SCCM. The flow rate of H_2_S gas was 65 SCCM.

The morphology of the samples was characterized via scanning electron microscopy (SEM, Hitachi S-4800). Their crystal structures were investigated by micro-Raman spectroscopy using an excitation band of 532 nm and a charge coupled device detector. Their optical property was characterized by UV–visible (UV–Vis) spectroscopy (Shimadzu UV-2600). PEC cells were fabricated on 1 × 2 cm^2^ ITO glass substrates. PEC characterization was performed using a three-electrode system with a Pt wire mesh as the working electrode and Ag/AgCl as the reference electrode. The electrolyte solution comprised 0.3 M KH_2_PO_4_ with KOH. The light source was a 150 W Xe arc lamp that delivers 100 mW/cm^2^ simulated AM 1.5 G irradiation. PEC measurements, including linear sweep voltammograms (LSVs) recorded using a sourcemeter (Keithley 2400), and electrochemical impedance spectroscopy (EIS) were conducted using an electrochemical analyzer (potentiostat/galvanostat 263A) in a three-electrode reactor. EIS analysis was performed at a bias of 0.6 V while varying the ac frequency from 100 kHz to 100 mHz. The IPCE of the electrode structure was measured using a grating monochromator within the excitation wavelength range of 300–800 nm. The hydrogen gas products were analyzed using a YL 6500 gas chromatograph (Young In Chromass, Republic of Korea) equipped with a flame ionization detector and a thermal conductivity detector.

## 3. Results

**Figure 1:**
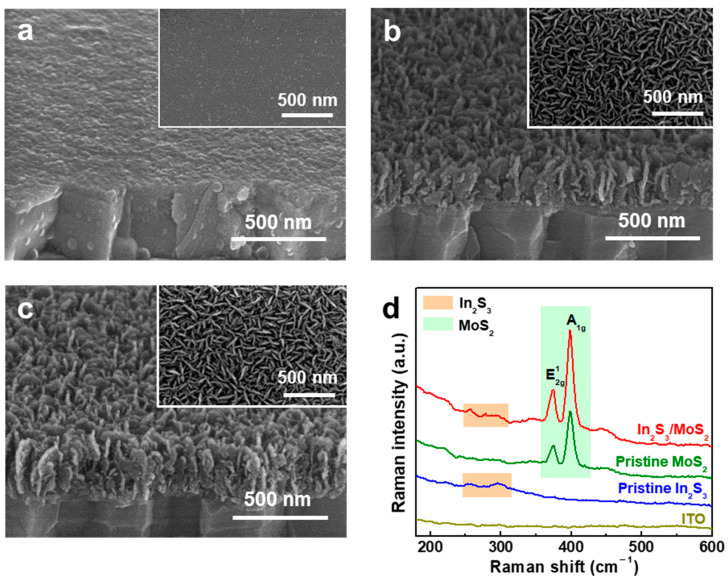
SEM images of (**a**) pristine In_2_S_3_, (**b**) pristine MoS_2_, and (**c**) In_2_S_3_/MoS_2_ and (**d**) Raman spectra of all films.

Figure 1a–c exhibit the top- and tilted-view SEM images of In_2_S_3_, 2D MoS_2_, and In_2_S_3_/MoS_2_. In_2_S_3_ possessed a layer of NPs on the ITO substrate with the thickness of ~50 nm (Figure 1a). This particle network resembled a uniform structure that acted as a seed layer for MoS_2_ growth. Vertically standing MoS_2_ NSs were uniformly generated on the ITO substrate (Figure 1b) and In_2_S_3_ (Figure 1c). The morphological characteristics of MoS_2_ on the entire surface of In_2_S_3_ appeared as vertically aligned NSs with a height of ~180 nm that developed by controlling the concentration ratio of Mo^4+^ to S^2−^ during the MOCVD reaction [11]. The adequate S^2−^ environment encouraged the growth of vertically-standing MoS_2_ NSs on In_2_S_3_.

The crystal structures of the samples (pristine In_2_S_3_, pristine MoS_2_, and In_2_S_3_/MoS_2_) were investigated by Raman spectroscopy. Our previous study revealed that the MoS_2_ NSs are few-layer 2D structures [2,11], which was also confirmed by Raman spectra (Figure 1d). In_2_S_3_ exhibited Raman peaks around 255 and 297 cm^–1^, corresponding to β-In_2_S_3_ [15], and two typical peaks of 2D-layered MoS_2_, corresponding to E^1^_2g_ and A_1g_ modes [16] for the in-plane vibration of S and Mo atoms and the out-of-plane vibration of S atoms, respectively. This finding indicates the successful growth of MoS_2_ NSs on In_2_S_3_.

**Figure 2:**
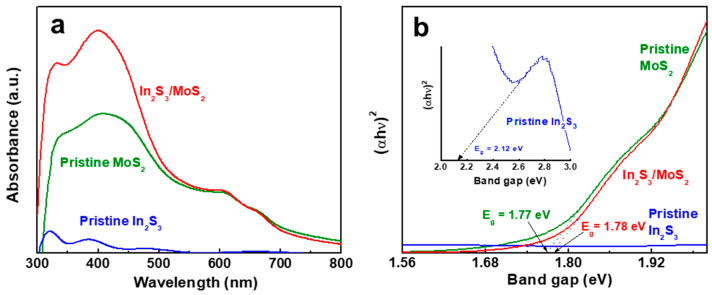
(**a**) UV–Vis absorption spectra and (**b**) Tauc plots of pristine In_2_S_3_, pristine MoS_2_, and In_2_S_3_/MoS_2_.

The optical properties evaluated by the UV–Vis absorbance analyses were strongly influenced by the presence of 2D MoS_2_ as shown in Figure 2a. Pristine MoS_2_ NSs exhibited an absorption edge of ~750 nm and two prominent absorption peaks at ~610 and ~665 nm, known as B and A excitons, respectively, which are correlated with direct excitonic transitions at the Κ point of the Brillouin zone [17]. Compared with pristine In_2_S_3_, the In_2_S_3_/MoS_2_ heterostructure showed improved absorbance attributed to the enhanced surface scattering of MoS_2_ 2D morphology. This result suggests a substantial improvement in the light absorption of the heterostructure with the decoration of MoS_2_ NSs. The optical bandgap energies (Figure 2b) calculated according to the Tauc equation [18] were 2.12 (In_2_S_3_), 1.77 (MoS_2_), and 1.78 eV (In_2_S_3_/MoS_2_) as estimated from the intercept of the linear portion of the Tauc plot. The similar bandgaps of MoS_2_ and In_2_S_3_/MoS_2_ amplified the ability of MoS_2_ for light absorption.

**Figure 3:**
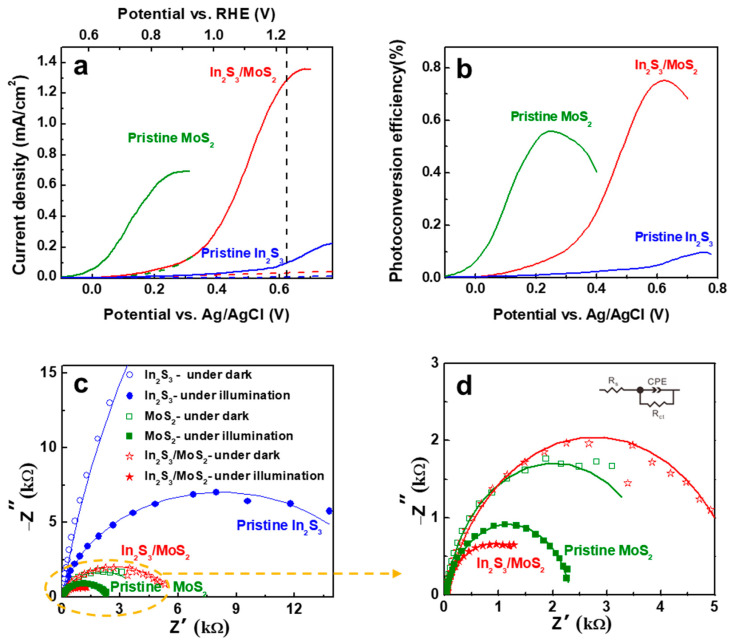
(**a**) Photo and dark current density−potential curves; (**b**) Photoconversion efficiency; and (**c**) Nyquist plots of PEC cells with pristine In_2_S_3_, pristine MoS_2_, and In_2_S_3_/MoS_2_. The yellow circle is enlarged in (**d**).

The PEC performance was evaluated by LSVs under simulation with AM 1.5 G illumination as depicted in Figure 3a. Compared with dark current curves, all the samples exhibited photocurrent attributed to the PEC reaction. The photocurrent density of pristine In_2_S_3_ was 0.097 mA/cm^2^, and that of In_2_S_3_ heterojunctioned with MoS_2_ was significantly improved up to 1.28 mA/cm^2^ at 1.23 V vs. RHE, which was higher than that of pristine MoS_2_ (0.85 mA/cm^2^ at 0.93 V vs. RHE). The enhanced PEC properties can be attributed to the effective electron–hole separation and transfer through the heterojunction.

Figure 3b shows the photoconversion efficiencies (η) of the samples estimated using the following equation [19]:

η = *J*(E_o_ − V_app_)/P_light_,

where *J* is the photocurrent density (mA/cm^2^) at the applied potential, E_o_ is the standard reversible potential (1.23 V), V_app_ is the applied potential, and P_light_ is the power density of illumination.

In_2_S_3_/MoS_2_ showed an η of 0.75% at 1.23 V vs. RHE which was substantially higher than that of pristine In_2_S_3_ (~0.1%). Figure 3c,d show the Nyquist plots of the EIS fitted using a simplified Randles circuit (inset in Figure 3d). In_2_S_3_/MoS_2_ exhibited smaller EIS semicircles, indicating a lower charge transfer resistance (R_ct_) of 1727 Ω under illumination than the pristine samples (16,350 Ω and 2308 Ω for In_2_S_3_ and MoS_2_, respectively). This result suggests that the heterojunction significantly improved the charge transfer efficiency.

**Figure 4:**
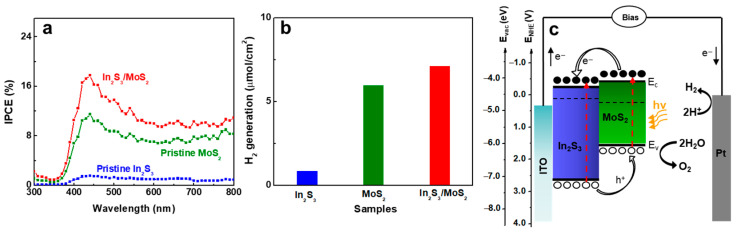
(**a**) IPCE plots and (**b**) hydrogen evolution amounts for 30 min of PEC cells with various working electrodes (pristine In_2_S_3_, pristine MoS_2_, and In_2_S_3_/MoS_2_) in 0.3 M KH_2_PO_4_ in KOH solution. (**c**) Schematic of the charge generation and transfer in the In_2_S_3_/MoS_2_ PEC cell.

A thorough study was performed using IPCE and H_2_ evolution to understand how the heterojunction enhanced the PEC performance. In_2_S_3_/MoS_2_ exhibited a peak value at ~440 nm and significant IPCE enhancement in the 600–750 nm region (Figure 4a), which was affected by the surface modulation with 2D MoS_2_ NSs. Hydrogen evolution from the dark cathode (Pt) was measured at 0.5 V versus Ag/AgCl using a three-electrode configuration for 30 min. The amount of produced H_2_ was significantly increased by the In_2_S_3_/MoS_2_ heterojunction as shown in Figure 4b, suggesting that the photocurrent was attributed to the WS. In_2_S_3_/MoS_2_ formed a staggered heterojunction (Figure 4c) [2,20], which was effective in separating and subsequently transferring photogenerated electrons and holes to the cathode (Pt electrode) through In_2_S_3_ and onto the anode (MoS_2_), leading to a boosted PEC performance.

**Figure 5:**
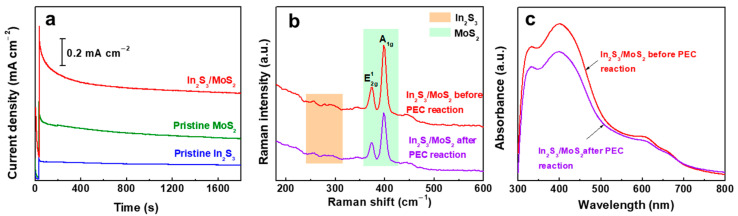
(**a**) Photocurrent–time plots for pristine In_2_S_3_, pristine MoS_2_, and In_2_S_3_/MoS_2_, and (**b**) Raman spectra and (**c**) UV–Vis absorption spectra of In_2_S_3_/MoS_2_ before and after PEC reaction.

Figure 5a shows the photocurrent density–time (J-t) curves of all of the photoanodes over 30 min. The photocurrent of In_2_S_3_/MoS_2_ stabilized after an initial decay period of ~400 s, which was similar to that of pristine MoS_2_. The initial photocurrent decay was attributed to recombination of the photogenerated holes with electrons [11]. After PEC reaction, the peak positions of Raman and UV–Vis absorption spectra of In_2_S_3_/MoS_2_ did not change, indicating no significant structural change. However, the full width at half maximum of Raman peaks slightly increased after reaction. Our recent study showed that MoS_2_ NSs are susceptible to subtle morphological changes due to the decomposition of MoS_2_, mainly the loss of S elements during PEC reaction [11].

## 4. Conclusions

In this study, 2D MoS_2_ NSs were vertically grown on a layer of In_2_S_3_ NPs using MOCVD. In_2_S_3_/MoS_2_ exhibited up to more than 13 times and 1.5 times higher photocurrent densities than pristine In_2_S_3_ and pristine MoS_2_, respectively, because of the extended visible-light absorption range and the efficient separation and transportation of the photogenerated carriers by the type-II heterojunction. The formation of a heterojunction with MoS_2_ NSs led to the maximum photoconversion efficiency of In_2_S_3_/MoS_2_ up to 0.75% at 1.23 V vs. RHE. This work suggests that the In_2_S_3_/MoS_2_ heterojunction is one of the feasible photoanodes for efficient PEC-WS.

## Data Availability

Data are available in the main text.
